# QRISK3 Assessment Identified Essential Thrombocythemia Patients at High Risk of Thrombotic Events Regardless of Revised IPSET‐T Score and JAK2 Mutational Status

**DOI:** 10.1002/jha2.70446

**Published:** 2026-10-28

**Authors:** Andrea Duminuco, Raj Vaghela, Sukhraj Virdee, Claire Woodley, Susan Asirvatham, Natalia Curto‐Garcia, Benissa Narciso, Priya Sriskandarajah, Jennifer O'Sullivan, Hugues De Lavallade, Deepti Radia, Elena Torre, Maria Georgiou, Shahram Kordasti, Giuseppe A. Palumbo, Claire Harrison, Patrick Harrington

**Affiliations:** ^1^ Dipartimento Di Scienze Mediche Chirurgiche e Tecnologie Avanzate “G.F. Ingrassia” University of Catania Catania Italy; ^2^ Department of Hematology Guy's and St Thomas' NHS Foundation Trust London UK; ^3^ School of Cancer and Pharmaceutical Science King's College London London UK

**Keywords:** cardiovascular risk factors, essential thrombocythemia, myeloproliferative neoplasms, polycythaemia vera, thrombosis

1

Thrombotic events (TEs) remain the most common cause of death among patients with myeloproliferative neoplasms (MPNs), including essential thrombocythemia (ET) and polycythemia vera (PV) [1]. As reported in several prospective trials, real‐world studies, and expert panel consensus, the dichotomy between low‐ and high‐risk classifications based on prior TE and age does not sufficiently define thrombotic risk. Our recently published study, based on the QRISK3 score, highlights the feasibility of using different approaches to identify high‐risk patients who could benefit from early cytoreductive treatment, and provides evidence for the pivotal role of cardiovascular risk factors in this risk assessment [2, 3, 4].

In patients with ET, the revised IPSET‐Thrombosis (IPSET‐T) score, which incorporates JAK status as well as thrombotic history and age, is commonly used to assess thrombotic risk. In PV, this is most typically based simply on age and thrombotic history, with some variation in the age threshold that is used to define a high‐risk population, with the BCSH guidelines for PV employing an age of > 65 years, however, in ELN and often in clinical practice, an age of > 60 defines the high‐risk group. Other biologic variables, such as leucocytosis and JAK2 V617F allele fraction, are also increasingly recognized as factors that should be considered in patients with PV when assessing thrombotic risk.

Here, we provide a more comprehensive analysis of the role of QRISK3, including the impact of MPN driver mutational status, and how it compares with current approaches, including the IPSET‐T score. We also performed additional analysis stratifying patients using an age cutoff of > 60 years to denote a high‐risk population, in contrast to our prior work which primarily used an age cutoff of 65 years [5]. The present analyses were performed in the same ET and PV cohorts previously reported in our original publication, in which the QRISK3 thresholds of 7.5% (ET and PV) and 5.5% (PV, ROC‐derived) were originally defined. As these cutoffs were derived and are here re‐applied within the same population, the reclassification frequencies and outcome differences reported below are not independent of the original findings and should not be interpreted as external validation of these thresholds.

In the ET cohort, the 490 patients are now stratified into 325 low‐ and 165 high‐risk, with the 30 patients falling between the ages of 60 and 64 years. The 46 TEs occurred in 26 cases in the low‐risk cohort and 20 in the high (8% vs. 12.1%, *p* = 0.14), showing no differences, albeit less marked than PV. Table 1 reports the full cross‐classification of conventional risk categories and QRISK3 strata (< 7.5% vs. ≥ 7.5%) for both the ET and PV cohorts, including TE counts and rates in each subgroup. Evaluating the QRISK3, again, it was higher in those who experienced a TE (2.8% vs. 10.1%, *p* < 0.001). Applying the 7.5% QRISK3 threshold [3], 50 (15.4%) low‐risk patients had a score higher than 7.5% and could be considered high‐risk (Table 1), in contrast to 97 (58.8%) high‐risk patients who reported a QRISK3 < 7.5%. Studying low‐risk patients, the TE was reported in 18 (36%) who had a QRISK3 ≥ 7.5%, with an occurrence higher than in patients with a score < 7.5% (8 TEs, 2.9%). Kaplan–Meier analysis confirmed reduced TFS in high QRISK3 patients (277 months), which differed statistically from that of those with a score < 7.5% (median survival not achieved, *p* < 0.001). Among the 50 conventional low‐risk QRISK3 ≥ 7.5% patients, 18 (36%) were on a watch‐and‐wait approach, with 8 of them experiencing a TE, and 32 (68%) underwent cytoreduction (with 10 reported TEs, *p* = 0.355).

**TABLE 1:** Risk class migration of conventional low‐risk patients according to standard scores and QRISK3 in essential thrombocythemia (A) and in polycythemia vera (B), allowing better stratification of patients.

Essential thrombocythemia (ET), *n* = 490			
		QRISK3 < 7.5%	QRISK3 ≥ 7.5%
Conventional low‐risk (age < 60, no prior TE)	*n* = 325	*n* = 275 TE = 8 (2.9%)	*n* = 50 TE = 18 (36.0%)
Conventional high‐risk (age ≥ 60 or prior TE)	*n* = 165	*n* = 97 TE = 8 (8.2%)	*n* = 68 TE = 12 (17.6%)
**Polycythemia vera (PV), *n* = 447**			
Conventional low‐risk (age < 60, no prior TE)	*n* = 290	*n* = 265 TE = 30 (11.3%)	*n* = 25 TE = 8 (32.0%)
Conventional high‐risk (age ≥ 60 or prior TE)	*n* = 157	*n* = 70 TE = 4 (5.7%)	*n* = 87 TE = 21 (24.1%)

Focusing specifically on driver mutations, 253 out of 424 patients with available data (59.7%) were JAK2‐mutated. Separating patients according to JAK2‐status and QRISK3 < or ≥ 7.5%, 4 risk classes were identified. TFS significantly differed according to QRISK3 score and JAK2 status (*p* < 0.001). Patients with high QRISK3 values (either JAK2‐mutated or wild‐type) showed markedly shorter TFS (median 277.1 and 193.1 months, respectively), whereas in both low‐QRISK3 groups median TFS was not reached (Figure 1A).

**FIGURE 1:**
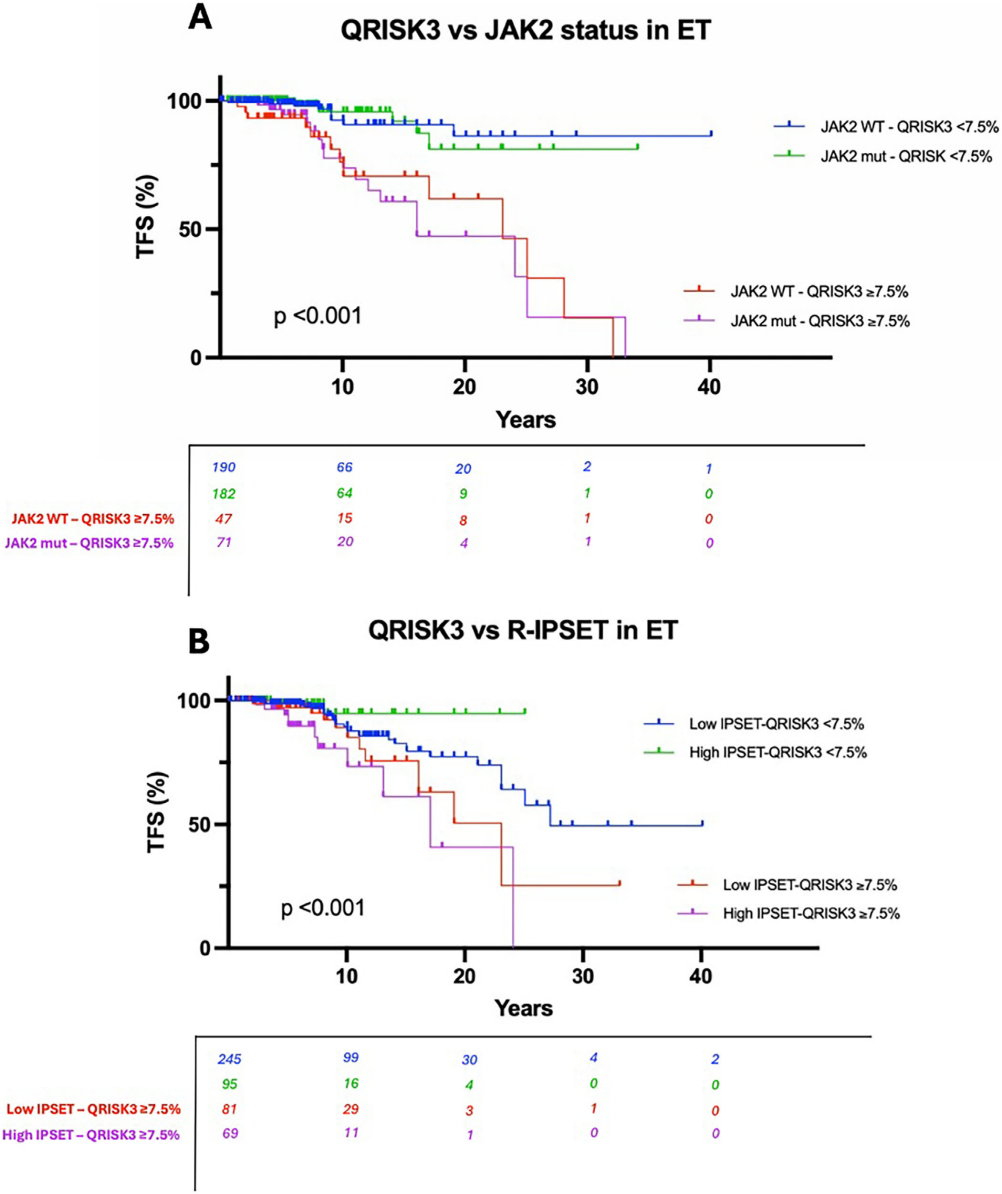
Stratification of thrombosis‐free survival in ET patients by QRISK3 score (≥ 7.5% vs. < 7.5%) combined with JAK2 mutational status (A) or revised‐IPSET risk category (B). QRISK3 further discriminated thrombotic risk within JAK2 and revised‐IPSET subgroups, whereas JAK2 status or revised‐IPSET category alone showed limited additional discrimination once QRISK3 was accounted for.

Similarly, we previously reported no significant difference in the proportion of ET patients experiencing thrombosis between those with very low and low IPSET‐T scores and those with intermediate and high risk, whereas adding QRISK3 evaluation in the very low and low risk groups improved this stratification [6, 7]. Here we expand on this using Kaplan–Meier analysis to assess TFS in these groups. Between TEs in those according to the IPSET‐T, ET patients were distributed into the four conventional risk categories: 138 very‐low (median TFS 385 months), 174 low (397 months), 21 intermediate (not reached), and 91 high (301 months; *p* = 0.04).

When grouping very‐low and low patients, who are not candidates for cytoreductive therapy, against intermediate and high‐risk patients, and further stratifying by QRISK3 < 7.5% or ≥ 7.5%, TFS differed significantly among subgroups (*p* < 0.001). Median TFS was 327 months for low IPSET‐T–QRISK3 < 7.5%, 277 months for low IPSET‐T–QRISK3 ≥ 7.5%, not reached for high IPSET‐T–QRISK3 < 7.5%, and 205 months for high IPSET‐T–QRISK3 ≥ 7.5%. Patients with high QRISK3 scores had the shortest TFS, highlighting the additive adverse effect of cardiovascular risk burden across IPSET‐T categories, rather than establishing superior discriminative power of QRISK3 over the IPSET‐T score itself, a comparison our analysis was not formally designed to test (Figure 1B).

Regarding PV patients, at diagnosis the cohort comprises 447 patients, of whom 290 are low risk, and 157 are high risk, with 22 patients being re‐categorized as high‐risk based on the lower age threshold. During follow‐up, the 62 TEs occurred in 24 high‐risk and 38 low‐risk patients (15.3% vs. 13.1%, *p* = 0.525). Conversely, the QRISK3 score was statistically higher for those who experienced TE (6.8% vs. 2.9%, *p* < 0.001).

Based on the QRISK3 thresholds described [3], 25 (8.6%) patients classified as standard low‐risk had a QRISK3 score ≥ 7.5%, and could be reclassified as high‐risk (Table 1), whereas 70 (44.6%) patients in the high‐risk group had a QRISK3 score lower than 7.5%. Focusing on the low‐risk subgroup, patients with QRISK3 ≥ 7.5% had reduced thrombosis‐free survival (TFS), reporting 8 TE during a median follow‐up of 228 months, which was statistically higher than that of those with QRISK3 < 7.5% (30 TE in 265 patients, median 303 months, *p* = 0.03). Among 12 patients managed with a watch‐and‐wait approach, 8 experienced TEs and 4 did not. In contrast, none of the 13 patients receiving cytoreductive therapy developed TEs (*p* < 0.001).

Expanding the analysis to a threshold of 5.5%, which receiver operating characteristic analysis showed was the optimal QRISK3 threshold in PV patients, the reclassified number of low‐risk patients increases to 65 (22.4%). The difference between low‐ and higher‐QRISK3 subgroups was more pronounced (mTFS not achieved vs. 173 months, *p* < 0.001). Numbers at risk and event counts for each of the four subgroups are reported below Figure 1 to allow assessment of the stability of these estimates, given the comparatively small number of patients and events in some strata.

Here, we again show that the QRISK3 score is a valuable adjunctive tool for refining cardiovascular risk stratification in patients with PV and ET. In ET patients, those with higher QRISK3 scores were at significantly increased risk of thrombosis regardless of JAK2 mutational status or IPSET‐T category, suggesting that QRISK3 can complement, rather than replace, existing risk‐stratification systems. These new findings also support its utility even when applying the widely adopted age threshold of 60 years to distinguish between high‐ and low‐risk categories in PV and ET.

Thrombosis in MPN is driven by both disease‐intrinsic mechanisms and conventional atherothrombotic risk factors, which are highly prevalent in this population and independently predict arterial events, explaining the validity of QRISK3 evaluation. In the international WHO‐ET cohort of 891 patients, cardiovascular risk factors (tobacco use, hypertension, or diabetes) were associated with an HR of 1.9 for arterial thrombosis, comparable in magnitude to JAK2 V617F (HR 2.6) and prior thrombosis (HR 2.1) [8]. QRISK3 integrates these modifiable contributors into a single, externally validated continuous metric, thereby capturing a dimension of risk only partially reflected by IPSET‐T and not by the age/history dichotomy used in PV [9].

While the history of prior TE remains a cornerstone of risk assessment, initiating cytoreductive therapy in patients under 60 years of age with a QRISK3 score ≥ 7.5% (or ≥ 5.5% in PV) and multiple cardiovascular risk factors may reduce thrombotic complications. Importantly, however, an elevated QRISK3 score is a potentially modifiable variable which should prompt systematic optimization of modifiable cardiovascular risk. Statins are of specific interest in this setting. In a SEER‐Medicare cohort of 4010 older patients with PV or ET, statin use was associated with improved overall survival and a reduced incidence of thrombosis, plausibly reflecting both lipid‐dependent and pleiotropic anti‐inflammatory and JAK2‐modulating effects [9, 10]. We would therefore frame QRISK3 not as a trigger for cytoreduction alone, but as an entry point to multidisciplinary cardiovascular risk management in MPN [11]. The apparent attenuation of IPSET‐T prognostic effect within the low‐QRISK3 subgroup should be interpreted cautiously given the limited number of patients and events in this stratum, and may reflect a chance finding rather than a true interaction between IPSET‐T and QRISK3.

These conclusions should be interpreted in light of several limitations. First, our analyses relied on Kaplan–Meier estimates and subgroup stratification rather than formal measures of comparative model performance, such as the C‐index, time‐dependent AUC, or net reclassification improvement (NRI); the relatively limited number of TEs (46 in ET, 62 in PV) precluded robust estimation of these metrics, and this should be regarded as a limitation rather than evidence of QRISK3's superiority over existing scores. Second, because QRISK3 was developed to predict overall cardiovascular events in the general population, and thrombosis in MPN comprises both arterial and venous events, its predictive value may be weighted toward arterial thrombosis; the limited number of events in our cohort did not allow a robust stratified analysis by thrombosis subtype, and this distinction should be addressed in future, adequately powered studies. Third, the retrospective design and the absence of randomized treatment allocation mean that the comparison between watch‐and‐wait and cytoreduction is subject to confounding by indication. Fourth, QRISK3 was derived and validated in a general UK primary‐care population rather than in patients with cancer or hematological malignancy; its calibration, discrimination, and clinical utility cannot be assumed to be preserved in MPN, where thrombotic risk is additionally driven by disease‐specific mechanisms such as JAK2 V617F allele burden, leucocytosis, and additional molecular abnormalities not captured by the score. We propose QRISK3 as complementary to, rather than a replacement for, these disease‐specific determinants; patients with discordant profiles (e.g., low QRISK3 but high molecular/biological risk, or vice versa) warrant individualized assessment integrating both dimensions. Finally, the principal finding that QRISK3 identifies MPN patients at increased thrombotic risk beyond conventional stratification has been reported previously by our group; the present analyses extend, but do not substantially change, that clinical message. External validation in independent MPN cohorts, incorporating formal comparative performance metrics, is therefore essential before QRISK3 can be recommended for routine thrombotic risk assessment or used to guide therapeutic decisions. Until such validation is available, the present findings should be regarded as confirmatory rather than practice‐changing.

## Author Contributions

Andrea Duminuco and Patrick Harrignton were responsible for: conceptualization, writing – original draft, methodology, validation, formal analysis, project administration, visualization, investigation, data curation. Raj Vaghela and Sukhraj Virdee were responsible for data curation and writing – review and editing. Claire Woodley, Susan Asirvatham, Benissa Narciso, Natalia Curto Garcia, Priya Sriskandarajah, Jennifer O'Sullivan, Hugues de Lavallade, Deepti Radia, Elena Torre, Maria Georgiou, Shahram Kordsati, Giuseppe Palumbo, and Claire Harrison were responsible for writing – review and editing.

## Funding

The authors have nothing to report.

## Conflicts of Interest

The authors declare no conflicts of interest.

## Data Availability

Data is available from the corresponding author on reasonable request.
